# Hydrogen therapy and cardiovascular diseases: a visualization analysis of research hotspots and application prospects

**DOI:** 10.4103/mgr.MEDGASRES-D-25-00165

**Published:** 2026-09-12

**Authors:** Yi Qian, Mingfei Shi, Yan Yang, Wei Qian, Ziyun Li, Jingqing Hu

**Affiliations:** 1 The Third Clinical Medical College, Nanjing University of Chinese Medicine, Nanjing, Jiangsu Province, China; 2 School of Integrated Chinese and Western Medicine, Nanjing University of Chinese Medicine, Nanjing, Jiangsu Province, China; 3 China Science and Technology Development Center for Chinese Medicine, Beijing, China; 4 School of Acupuncture and Tuina, School of Regimen and Rehabilitation, Nanjing University of Chinese Medicine, Nanjing, Jiangsu Province, China; 5 School of Traditional Chinese Medicine, Tianjin University of Traditional Chinese Medicine, Tianjin, China

**Keywords:** anti-inflammatory, anti-oxidant, bibliometric analysis, cardiovascular disease, hydrogen therapy, ischemia-reperfusion injury, molecular hydrogen, NLRP3 inflammasome, research hotspots, visualized analysis

## Abstract

Hydrogen, an emerging medical gas, has demonstrated diverse biological effects, including anti-oxidant, anti-inflammatory, and anti-apoptotic activities, offering significant therapeutic potential in cardiovascular diseases. This study aimed to explore the applications of hydrogen therapy in cardiovascular diseases and to illustrate both the current research status and future trends. 162 publications (129 original articles and 33 reviews) on hydrogen therapy in cardiovascular diseases were retrieved from the Web of Science Core Collection database up to April 9, 2025. The information was analyzed using Excel 2019, OriginPro 2024, VOSviewer 1.6.20 and CiteSpace 6.2. The number of publications on hydrogen therapy for cardiovascular diseases has shown a significant increase, with China, Japan, and the United States of America being the leading contributors. Keio University published the largest number of articles, and Sano Motoaki, the most prolific author (*n* = 14), is also from Keio University. Keyword clustering analysis shows that current research hotspots include “ischemia-reperfusion injury,” “cardiac arrest,” “oxidative stress,” “inflammation,” and “apoptosis,” while emerging directions may focus on the “NLRP3 inflammasome,” “pyroptosis,” and “micro/nanomaterials.” The underlying mechanisms of hydrogen therapy and its delivery approaches remain key research hotspots of hydrogen therapy for cardiovascular diseases; however, further high-quality clinical studies are needed to strengthen the evidence base and support its wider clinical application.

## Graphical abstract

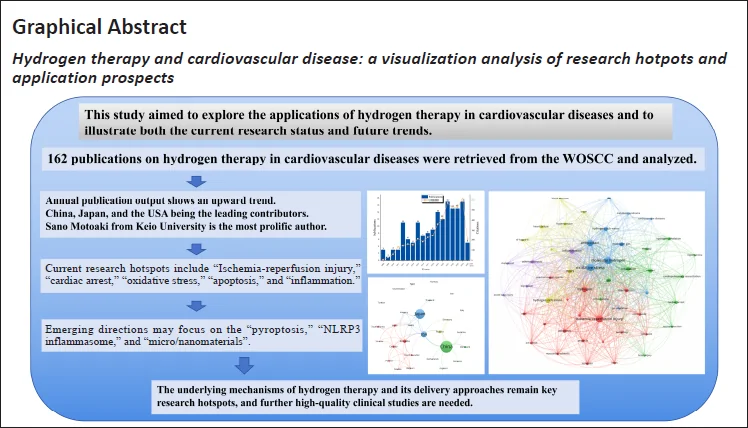

## Introduction

Molecular hydrogen is a colorless, odorless gaseous molecule with the smallest molecular weight among all gas molecules. It exhibits various biological effects, such as anti-oxidant activity and is recognized as a novel medical gas molecule.^1^ In 2007, Shigeo Ohta demonstrated that inhalation of low-concentration (2–4%) hydrogen significantly reduced brain damage caused by cerebral ischemia–reperfusion (I/R) in rats through its anti-oxidant activity, catalyzing rapid advances in hydrogen biomedicine.^2^ As research has progressed, additional mechanisms of molecular hydrogen have been identified, including anti-inflammatory, anti-apoptotic, autophagy and regulation of various cellular pathways.^3^ Clinical trials have further demonstrated the safety and efficacy of molecular hydrogen in multiple conditions, including post-cardiac arrest syndrome, unstable angina pectoris, acute cerebral infarction, non-alcoholic fatty liver disease, metabolic syndrome, and chronic obstructive pulmonary disease.^4^,^5^,^6^,^7^,^8^,^9^

Owing to its superior safety profile compared with other biologically active gases, molecular hydrogen holds great potential for disease prevention and treatment. Moreover, molecular hydrogen can be easily absorbed by the human body and delivered through various routes, such as inhalation of hydrogen gas, ingestion of hydrogen-rich water, injection or topical administration of hydrogen-rich saline to the surface, and hydrogen water baths.

Although several clinical studies have confirmed the potential efficacy of hydrogen therapy for cardiovascular diseases, its clinical application remains limited.^10^ Controversy persists regarding both the efficacy and safety of hydrogen therapy, and the future development of this field remains unclear. To address this, we used literature visualization and bibliometric analysis methods to summarize the development trends, research hotspots, and prospects of hydrogen therapy for cardiovascular diseases, aiming to provide a reference for its clinical application.

## Methods

### Data source and retrieval strategy

Data were retrieved from the Web of Science Core Collection database on April 9, 2025. Search keywords included “cardiovascular diseases,” “molecular hydrogen,” “hydrogen therapy,” “hydrogen-rich saline,” “hydrogen gas,” and “hydrogen-rich water” (**Additional file 1**). The document types were limited to the original articles and reviews and the language of literature was limited to English. Only officially published papers and reviews were included; preprints were excluded to minimize publication bias. No restrictions were applied regarding the publication period.

### Data extraction and statistical analysis

The included publications were exported in BibTeX format and compiled in Excel 2019 (Microsoft, Redmond, WA, USA). The dataset contained information on authors, keywords, institutions, abstracts, sources, citation counts, titles, article types, and publication years. Data analysis was performed using OriginPro 2024 (OriginLab Corporation, Northampton, MA, USA), CiteSpace 6.2 (Drexel University, USA) and VOSviewer 1.6.20 (Center for Science and Technology Studies, Leiden University, the Netherlands). VOSviewer was used to visualize a network of journals and create bibliographic, co-citation or co-authorship maps, enabling analysis of literature trends and patterns.^11^ To minimize bias arising from different expressions related to the same author or keywords, the data were manually standardized before visualization.^12^ CiteSpace was used to conduct co-citation analysis and detect citation bursts of authors, keywords and references. The CiteSpace parameters were set as follows: time slicing (from January 2008 to May 2025 with a slice length of 1 year), selection criteria using the g-index (k = 25), and pruning via Minimum Spanning Tree and Pruning Sliced Networks.

## Results

### Annual growth trend of publications and citations of research about the hydrogen therapy for cardiovascular diseases

A total of 162 relevant publications were identified, comprising 129 articles and 33 reviews (**Figure 1**). The earliest research on hydrogen therapy for cardiovascular diseases was published in 2008. Of the 162 publications, 10 (6.2%) were published between 2008 and 2011, 57 (35.2%) between 2012 and 2018, and 95 (58.6%) between 2019 and April 2025. The peak publication years regarding this topic were 2021 and 2024. The total number of annual citations steadily increased over time.

**Figure 1:**
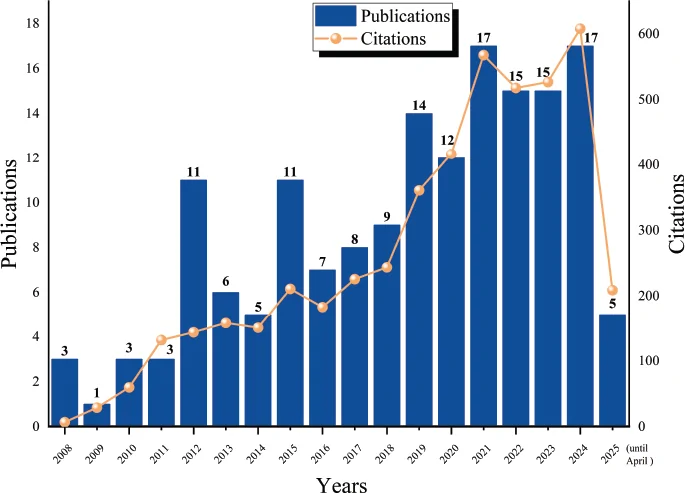
Annual number of publications and citations of research about the hydrogen therapy for cardiovascular diseases. Data were collected from Web of Science Core Collection Database on April 9, 2025. Created with OriginPro 2024.

### Collaboration between countries/institutions of research about the hydrogen therapy for cardiovascular diseases

319 institutions from 28 countries were involved in total. Countries with three or more publications and the top 10 affiliations are shown in **Figure 2A** and **B**. China contributed the largest number of publications (*n* = 78), followed by Japan (*n* = 53) and the USA (*n* = 21). Keio University led in publications within this field (*n* = 17), followed by Naval Medical University with 13 publications. **Figure 3** illustrates strong international collaboration, particularly between China, Japan, the USA, and Slovakia. The study revealed that the USA has the most extensive collaborative network, with a total link strength of 21, followed by Slovakia (17) and China (15). Among these 319 institutions, 241 institutions (75.5%) contributed to only one study, while 61 (19.1%) were involved in two to three studies, 13 institutions (4.1%) participated in four to nine studies, and four (1.3%) contributed to more than ten studies.

**Figure 2:**
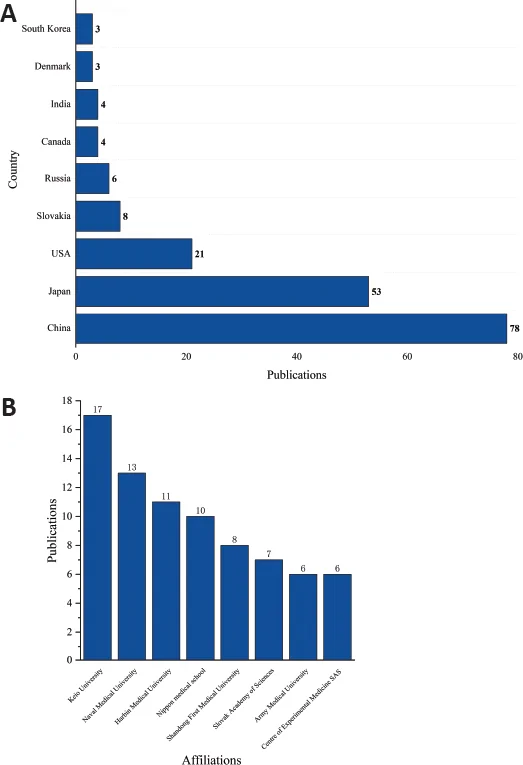
The countries and affiliations of publications related to hydrogen therapy for cardiovascular diseases. (A)The countries of publications. (B) The affiliations of publications. Created with OriginPro 2024.

**Figure 3:**
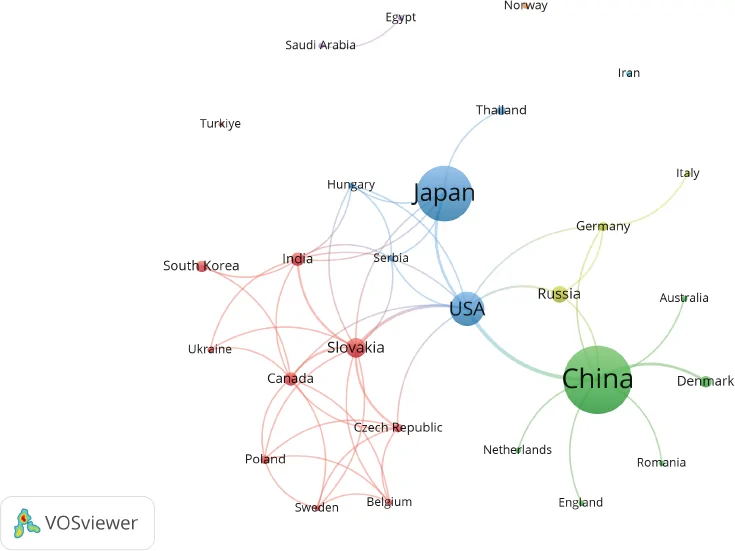
The international cooperation on hydrogen therapy for cardiovascular diseases. The size of each circle represents the number of publications. Line thickness indicates collaboration strength between nodes. Created with VOSviewer 1.6.20.

### Authorship collaboration of research about the hydrogen therapy for cardiovascular diseases

A total of 942 authors participated in the research field, of whom 766 (81.32%) authored a minimum of one study. In addition, 113 authors published two studies, 25 published three, 19 published four, and 19 authors published at least five. Sano Motoaki emerged as the leading author with 14 publications and 1005 citations, surpassing all other contributors by a notable margin. He was followed by Suzuki Masaru, who authored 9 publications with 411 citations, and Wei Yang, who authored 9 publications and 208 citations (**Table 1**). **Figure 4** depicts the collaborative network involving 38 authors, each having published at least four documents. Analysis identified seven distinct research teams, with the top 10 authors distributed across only three clusters, six of whom belonged to the same team.

**Figure 4:**
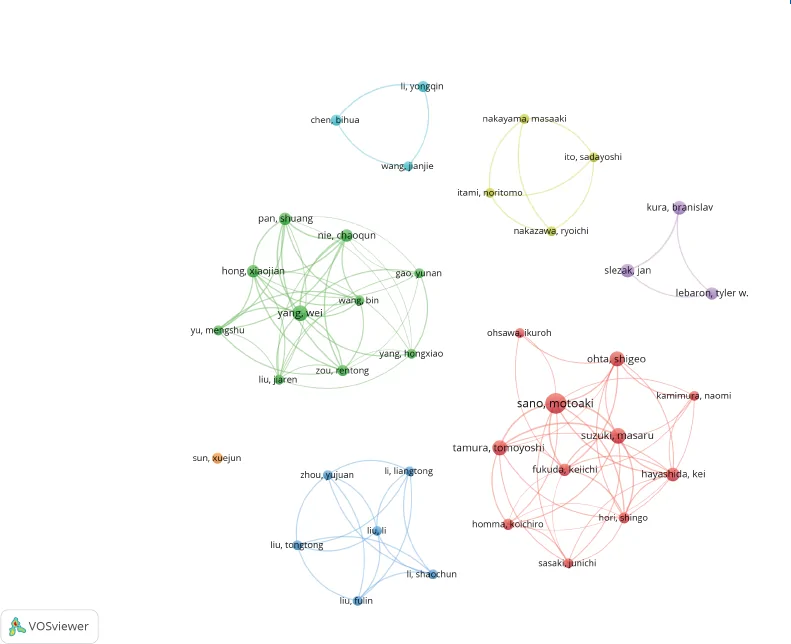
The author collaborative network on hydrogen therapy for cardiovascular diseases. The size of each circle represents the number of publications published by the authors. The lines connecting two circles indicates collaboration between those authors. Created with VOSviewer 1.6.20.

**Table 1:** Top 10 authors in hydrogen therapy for cardiovascular diseases

Author	Institution	*n*	Citations
Sano Motoaki	Keio University (Japan)	14	1005
Suzuki Masaru	Keio University (Japan)	9	411
Wei Yang	Harbin Medical University (China)	9	208
Ohta Shigeo	Nippon Medical School (Japan)	8	992
Tamura Tomoyoshi	Keio University (Japan)	8	248
Hayashida Kei	Keio University (Japan)	7	323
Kura Branislav	Slovak Academy of Sciences (Slovak)	7	249
Slezak Jan	Slovak Academy of Sciences (Slovak)	7	249
Fukuda Keiichi	Keio University (Japan)	6	789
Xiaojian Hong	Harbin Medical University (China)	6	83
Lebaron Tyler W.	Slovak Academy of Sciences (Slovak)	6	213
Chaoqun Nie	Harbin Medical University (China)	6	135
Pan Shuang	Harbin Medical University (China)	6	135

### Keywords mapping of research about the hydrogen therapy for cardiovascular diseases

A total of 917 keywords were extracted, with a cumulative frequency of 2005 occurrences. **Table 2** displays the top 20 keywords associated with hydrogen therapy in cardiovascular diseases. The top 25 most-cited keywords highlight the research frontiers. The keywords with citation bursts included myocardial ischemia, endoplasmic reticulum stress and apoptosis during the early years (2008–2018); from 2019 to 2025, the strongest citation bursts were observed for hydrogen-rich water, cardiac arrest, NOD-like receptor family pyrin domain containing 3 (NLRP3) inflammasome, pyroptosis and clinical trials (**Figure 5**). Cluster analysis was applied to keywords occurring ≥ 6 times, identifying five distinct clusters. In the blue I cluster, frequently occurring keywords included oxidative stress, molecular hydrogen, anti-oxidant, hydrogen-rich water, myocardial infarction, etc. The red II cluster focused on I/R injury, hydrogen, apoptosis, mechanisms, etc. The main keywords in the green III cluster included model, inhalation, gas, cardiac arrest, etc. In the yellow IV cluster, dominant keywords were hydrogen-rich saline, injury, heart failure, hypertension, etc. The purple V cluster centered on inflammation, atherosclerosis, expression, cholesterol, etc. (**Figure 6**).

**Figure 5:**
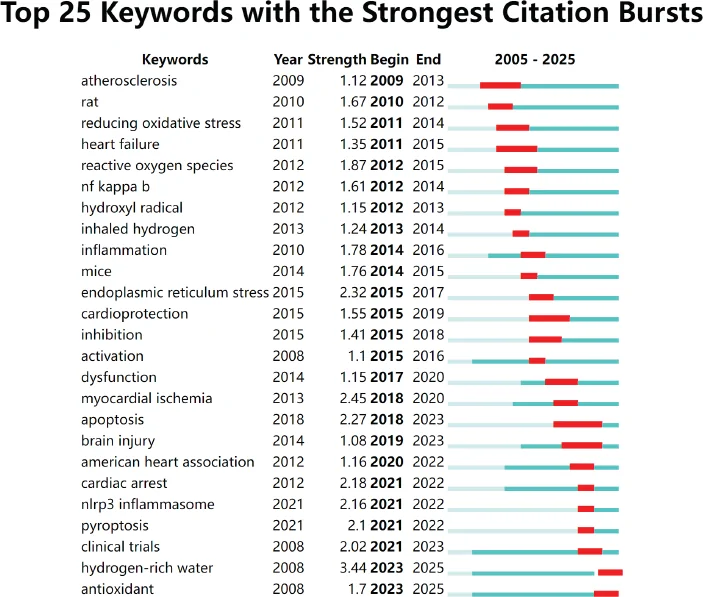
The top 25 keywords with the strongest citation bursts of hydrogen therapy for cardiovascular diseases. Created with CiteSpace 6.2. nlrp3: NOD-like receptor family pyrin domain containing 3; nf kappa b: nuclear factor kappa-B.

**Figure 6:**
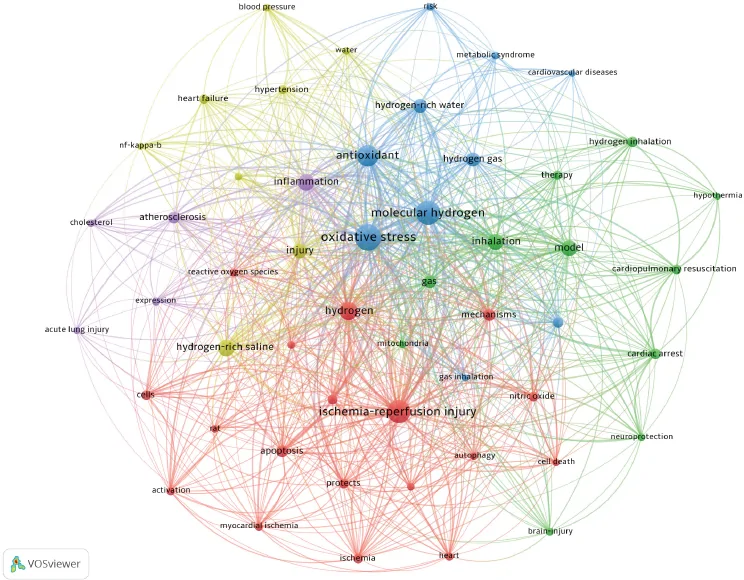
The co-occurrence network of the keywords in hydrogen therapy for cardiovascular diseases. The size of nodes represents keyword frequency, while thicker lines indicate increased co-occurrence among keywords. Colors represent keyword clustering. Created using VOSviewer 1.6.20.

**Table 2:** Top 20 keywords in hydrogen therapy for cardiovascular diseases

Rank	Keyword	*n*
1	Oxidative stress	74
2	Molecular hydrogen	62
3	Ischemia-reperfusion injury	56
4	Antioxidant	52
5	Hydrogen	37
6	Inflammation	32
7	Hydrogen-rich saline	31
8	Inhalation	30
8	Model	30
10	Injury	24
11	Hydrogen-rich water	23
12	Hydrogen gas	22
13	Gas	19
14	Apoptosis	17
15	Mechanisms	16
15	Atherosclerosis	16
17	Myocardial infarction	14
18	Cardiac arrest	13
18	Hydrogen inhalation	13
20	Heart failure	12

### Co-cited references and citation bursts of research about the hydrogen therapy for cardiovascular diseases

We present the top 10 co-cited references, which consist of eight original articles and two reviews (**Additional Table 1**). Four of these references were co-cited more than 30 times in total. These studies summarized that hydrogen therapy is effective for cardiovascular diseases such as myocardial I/R injury, and cardiac arrest. Ohsawa’s study,^2^ published in *Nature Medicine* in 2007, has received the highest number of co-citations.

**Additional Table 1:** Top 10 co-cited references relevant to hydrogen therapy for cardiovascular diseases

Rank	Co-cited reference	Co-citation
1	Ohsawa I, Ishikawa M, Takahashi K, et al. Hydrogen acts as a therapeutic antioxidant by selectively reducing cytotoxic oxygen radicals. *Nat Med*. 2007;13:688-694.	134
2	Hayashida K, Sano M, Ohsawa I, et al. Inhalation of hydrogen gas reduces infarct size in the rat model of myocardial ischemia-reperfusion injury. *Biochem Biophys Res Commun*. 2008;373:30-35.	68
3	Ohta S. Molecular hydrogen as a preventive and therapeutic medical gas: initiation, development and potential of hydrogen medicine. *Pharmacol Ther*. 2014;144:1-11.	30
4	Hayashida K, Sano M, Kamimura N, et al. H(2) gas improves functional outcome after cardiac arrest to an extent comparable to therapeutic hypothermia in a rat model. *J Am Heart Assoc*. 2012;1:e003459.	29
5	Hayashida K, Sano M, Kamimura N, et al. Hydrogen inhalation during normoxic resuscitation improves neurological outcome in a rat model of cardiac arrest independently of targeted temperature management. *Circulation*. 2014;130:2173-2180.	26
5	Ohta S. Recent progress toward hydrogen medicine: potential of molecular hydrogen for preventive and therapeutic applications. *Curr Pharm Des*. 2011;17:2241-2252.	26
7	Kajiyama S, Hasegawa G, Asano M, et al. Supplementation of hydrogen-rich water improves lipid and glucose metabolism in patients with type 2 diabetes or impaired glucose tolerance. *Nutr Res*. 2008;28:137-143.	25
7	Ohsawa I, Nishimaki K, Yamagata K, Ishikawa M, Ohta S. Consumption of hydrogen water prevents atherosclerosis in apolipoprotein E knockout mice. *Biochem Biophys Res Commun*. 2008;377:1195-1198.	25
9	Nakao A, Toyoda Y, Sharma P, Evans M, Guthrie N. Effectiveness of hydrogen rich water on antioxidant status of subjects with potential metabolic syndrome-an open label pilot study. *J Clin Biochem Nutr*. 2010;46:140-149.	24
9	Sun Q, Kang Z, Cai J, et al. Hydrogen-rich saline protects myocardium against ischemia/reperfusion injury in rats. *Exp Biol Med (Maywood)*. 2009;234:1212-1219.*	24

*This article was retracted.

**Figure 7A** illustrates the top 15 references across each year slice generated by CiteSpace. Two papers demonstrated the strongest bursts, lasting 4 and 5 years, respectively, both focusing on the molecular mechanisms underlying hydrogen therapy.

**Figure 7:**
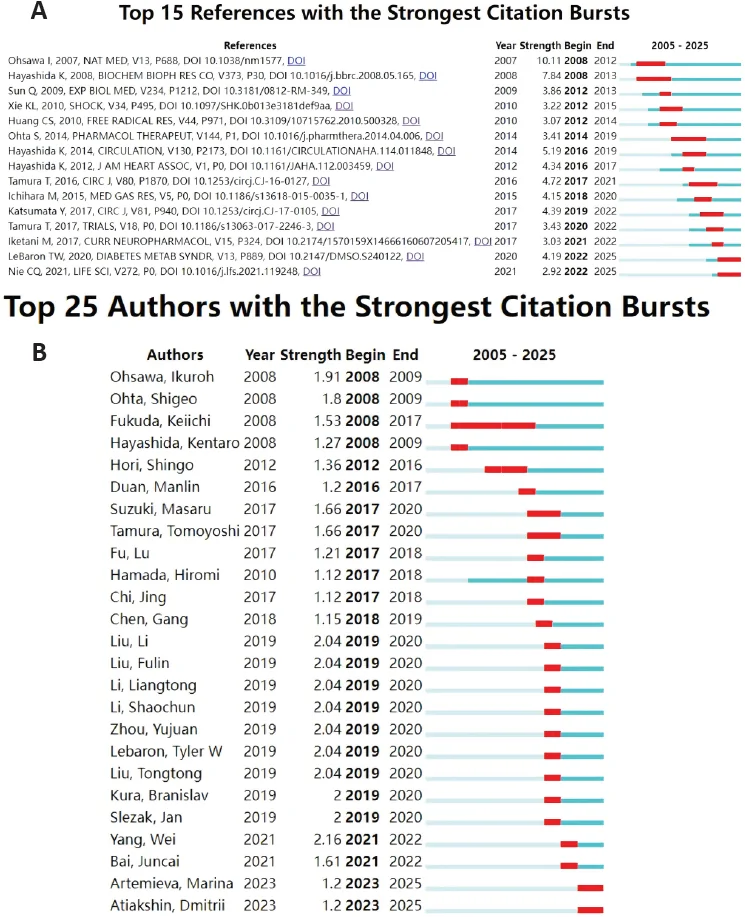
The top 15 references and top 25 authors with the strongest citation bursts of research about the hydrogen therapy for cardiovascular diseases. (A)The top 15 references with the strongest citation bursts. (B) Top 25 authors with the strongest citation bursts. Created with CiteSpace 6.2.

**Figure 7B** reveals the top 25 co-cited authors with bursts ≥ 1 year. Such burst patterns may signify periods of active development, indicating that these references and authors represent the forefront of research on hydrogen therapy for cardiovascular diseases.

## Discussion

Bibliometric analysis, as a quantitative methodology, assesses key characteristics and scholarly impact of highly cited studies. In this study, we employed CiteSpace and VOSviewer to analyze the literature on hydrogen therapy for cardiovascular diseases indexed in the Web of Science Core Collection database. The visualization through knowledge graphs enables researchers to quickly grasp research hotspots and evolution trends in the field, identify shifts in research focus across different periods, and facilitate a more intuitive understanding and formulation of future research plans.

Recent years have witnessed a significant expansion of hydrogen therapy applications in cardiovascular diseases. Analysis of country and institutional contributions indicates that China, Japan, and the USA are the major contributors in this field. China, in particular, dominates research on hydrogen therapy for cardiovascular diseases, accounting for approximately half of the global publications, reflecting its strong research capabilities and innovation potential. However, Japan’s citation count substantially exceeds that of China, with six institutions ranking among the top 10 by citation. Japan has played a pioneering role in early basic research, indicating its high research level and international recognition. The USA functions as the central hub for global collaboration. Thus, while China leads in quantitative output, Japan exerts qualitative influence through foundational contributions and high citation impact, and the USA facilitates international cooperation.

Sano Motoaki from Keio University emerged as the most prolific author among the top 10 contributors. Cluster analysis revealed that six of these authors belonged to a single cluster, while the remaining four maintained separate collaborative networks. **Figure 4** illustrates the structural independence between these clusters, indicating that substantial cross-group collaborations have not yet been developed and that research networks remain relatively independent in their patterns of academic cooperation.

Keywords provide real-time insights into a research field, reflect current hotspots and development trends, and help researchers understand the forefront of the discipline and its knowledge structure. **Table 2** shows the high-frequency keywords in the hydrogen therapy applications for cardiovascular diseases, including oxidative stress, molecular hydrogen and I/R injury. Cluster analysis of keyword networks identified five clusters: (I) myocardial infarction, (II) I/R injury, (III) cardiac arrest, (IV) hypertension/heart failure, and (V) atherosclerosis.

Myocardial infarction is the pathological process of myocardial cell necrosis caused by myocardial ischemia and hypoxia. Restoration of blood flow after myocardial infarction is necessary, but it may also induce I/R injury, leading to serious consequences such as myocardial stunning and reperfusion arrhythmia.^13^ In 2008, Hayashida et al.^14^ found that inhalation of hydrogen gas improved left ventricular function in isolated hearts after hypoxia reoxygenation, and reduced infarct size and left ventricular remodeling in rats with myocardial I/R injury. Proteomic and bioinformatics analyses suggest that hydrogen gas may alleviate myocardial I/R injury by modulating pathways involved in cell apoptosis, energy metabolism, and inflammatory response.^15^ Further studies proved that hydrogen-rich water inhibits myocardial cell apoptosis and reduces oxidative stress levels by activating the phosphatidylinositol 3-kinase/protein kinase B, Janus kinase 2/signal transducer and activator of transcription 3 and nuclear factor erythroid 2-related factor 2/anti-oxidant response element signaling pathway in myocardial I/R injury rats.^16^,^17^ Another study showed that hydrogen-rich water reduced cardiac inflammatory markers such as neutrophil infiltration, 3-nitrotyrosine level, expression of intercellular adhesion molecule 1 and myeloperoxidase activity, in the area-at-risk zones of rat hearts subjected to regional myocardial I/R injury. It also attenuated the I/R-induced elevation of proinflammatory cytokines, including tumor necrosis factor α and interleukin 1β.^18^ Hydrogen inhalation also inhibited apoptosis induced by reactive oxygen species-mediated mitochondrial-dependent pathway, thereby alleviating acute myocardial infarction in rats.^19^ From 2016 to 2018, as the understanding of pyroptosis deepened, numerous mechanisms underlying cardiovascular pathogenesis were gradually elucidated, such as the activation of NLRP3 inflammasome as a novel pathway of myocardial injury. Nie et al.^20^,^21^ found that inhalation of hydrogen gas reduced myocardial infarction in rats by inhibiting oxidative stress and NLRP3-mediated pyroptosis. Furthermore, in vitro experiments revealed that molecular hydrogen not only reduced hypoxia-induced cardiomyocyte damage but also suppressed the migration and activation of cardiac fibroblasts, thereby ameliorating myocardial remodeling and fibrosis following myocardial infarction. Molecular hydrogen also inhibits apoptosis by suppressing the endoplasmic reticulum stress pathway.^22^ Beyond hydrogen gas and hydrogen-rich water, a hydrogen gas perfluorooctyl bromide nanoemulsion with high hydrogen-loading capacity has been developed for targeted precision therapy of ischemic myocardium. This nanoemulsion effectively enables selective infiltration of the ischemic myocardium and real-time visualization using ^19^fluorine magnetic resonance imaging system, thereby exerting potent anti-oxidant and anti-inflammatory effects against myocardial I/R injury.^23^ Katsumata et al.^24^ treated 20 patients with ST-elevated myocardial infarction with 1.3% hydrogen gas for 6 months. The results showed that low-concentration hydrogen gas treatment after myocardial infarction promoted left ventricular reverse remodeling without detectable toxic side effects, although larger-scale studies are still required for validation. A clinical trial conducted by Si et al.^5^ in 2021 showed that consumption of 1–1.2 L of hydrogen-rich water daily for 3 consecutive months alleviated angina symptoms in patients with unstable angina and reduced plasma levels of total cholesterol, low-density lipoprotein cholesterol, and apolipoprotein B.

Cardiac arrest, one of the most common and lethal acute cardiovascular events, leads to death or permanent neurological deficits in approximately 75% of patients despite successful restoration of spontaneous circulation after cardiopulmonary resuscitation.^25^ Post-cardiac arrest syndrome involves clinical manifestations that primarily result from I/R injury in multiple organ systems, notably the cerebral and cardiac systems.^26^ Hayashida et al.^27^,^28^ pioneered the investigations into hydrogen therapy in cardiac arrest by establishing an animal model and demonstrating that inhalation of hydrogen gas improved post-resuscitation survival and mitigated neurological deficits in rats following cardiopulmonary resuscitation. Furthermore, studies revealed that the therapeutic effects of hydrogen are primarily attributed to its anti-oxidant, anti-inflammatory, and anti-apoptotic properties, inhibition of autophagy, upregulation of regulatory T cells and reduction of endothelial damage.^29^,^30^,^31^,^32^ In 2016, Tamura et al.^4^ published a clinical study in which five comatose patients who had restoration of spontaneous circulation after cardiac arrest received continuous inhalation of 2% hydrogen gas for 18 hours. Four of the five patients survived for 90 days with improved neurological outcomes, whereas one patient died of pneumonia-induced septic shock 22 hours after discontinuation of the trial (deemed unrelated to hydrogen gas inhalation by the hospital ethics committee). Despite its small cohort, this pilot study provided the first clinical evidence supporting the potential neuroprotective effects of hydrogen gas inhalation in post–cardiac arrest patients. Notably, Japan authorized the emergency clinical use of 2% hydrogen gas inhalation for cardiac arrest in the same year. Afterwards, Tamura et al.^33^ conducted a prospective, multicenter, randomized, double-blind, placebo-controlled trial involving 73 comatose patients following cardiogenic out-of-hospital cardiac arrest. Patients were randomly assigned in a 1:1 ratio using blinded gas cylinders to receive either supplementary oxygen with 2% hydrogen gas or oxygen for 18 hours. The results showed that, although hydrogen inhalation did not significantly improve the proportion of patients with out-of-hospital cardiac arrest achieving favorable neurological outcomes, it improved the 90-day survival rate and modified Rankin scale scores. However, further validation with larger sample sizes is warranted.

The progression of hypertension and heart failure is closely related to the persistent inflammatory responses and oxidative stress within the vasculature and myocardium. Studies showed molecular hydrogen did not significantly affect blood pressure in rats, but alleviated vascular dysfunction, reduced myocardial fibrosis, and attenuated left ventricular hypertrophy.^34^,^35^ However, another study showed that hydrogen significantly reduced blood pressure and corrected abnormal vascular relaxation in hypertensive rats induced by intermittent hypoxia.^36^ This difference may be attributed to differences in methods used to establish hypertension models. A large retrospective study conducted in 2024 (*n* = 2364) showed that, in adult hypertensive patients receiving conventional treatment, combined hydrogen inhalation further reduced blood pressure and was well tolerated.^37^ Molecular hydrogen also attenuates the progression of pulmonary hypertension.^38^,^39^,^40^ As hypertension can gradually progress to heart failure, molecular hydrogen may help prevent the development of congestive heart failure by inhibiting p53-mediated apoptosis.^41^

The pathogenesis of atherosclerosis is fundamentally driven by lipid metabolism disorders. Accumulating evidence indicates that manifestations of metabolic syndrome, including hypertension, dyslipidemia, hyperglycemia, and hyperuricemia, are critical upstream contributors to the pathogenesis and progression of atherosclerosis.^42^ In 2008, Ohsawa et al.^43^ reported that hydrogen-rich saline significantly reduced the formation of atherosclerotic plaques in apolipoprotein E knockout mice. Subsequent studies have proved that molecular hydrogen regulates lipid metabolism disorders and inhibits atherosclerosis progression through its anti-oxidation and anti-inflammatory effects.^44^,^45^,^46^ Song et al.^47^,^48^ found that hydrogen-rich water reduces the levels of serum total cholesterol and low-density lipoprotein cholesterol, enhances high-density lipoprotein anti-atherosclerotic functions, and has beneficial lipid-lowering effects. LeBaron et al.^49^ conducted a randomized, double-blind, placebo-controlled trial in 60 subjects with metabolic syndrome. The results showed that 24 weeks of high-concentration hydrogen-rich water significantly reduced blood cholesterol and glucose levels and lowered serum hemoglobin A1c in metabolic symptom patients, suggesting a potential role in modulating oxidative stress and inflammatory pathways.

In recent years, hydrogen therapy has been increasingly used for the treatment of cardiovascular diseases. The mechanisms underlying the anti-inflammatory, anti-oxidant, and anti-apoptotic effects of molecular hydrogen have consistently remained a central focus of molecular hydrogen research. As shown in **Figure 5**, NLRP3 and pyroptosis have emerged as key research frontiers in elucidating the underlying mechanisms of hydrogen therapy, accompanied by a growing number of clinical trials investigating its applications in cardiovascular diseases. Beyond mechanistic and clinical studies, hydrogen delivery methods have been continuously evolving. The application of micro/nanomaterials has significantly enhanced the therapeutic efficacy of hydrogen therapy by enabling targeted hydrogen delivery, controlled hydrogen release, and nanocatalytic and multimodal enhancement of its efficacy.^21^,^50^,^51^ Although a number of studies have demonstrated the beneficial effects of hydrogen therapy for cardiovascular diseases, its underlying mechanisms and optimal delivery strategies remain critical topics. Furthermore, clinical trials in this field remain limited, often constrained by small sample sizes and insufficient investigation of dose-response relationships. High-quality clinical evidence is required to facilitate its broader clinical application.

The research still has certain limitations: (1) The analysis was conducted exclusively on the Web of Science Core Collection database, excluding publications from other databases and non-English studies, which may introduce selection bias in the literature pool. (2) To ensure the reliability of the results, manual standardization was performed to reduce conceptual inconsistencies arising from different expressions of the same concepts. Nevertheless, such errors can only be minimized rather than entirely eliminated. (3) The relatively small number of eligible literatures limit the generalizability of the findings.

This study conducted a bibliometric analysis to summarize and evaluate the literature on hydrogen therapy for cardiovascular diseases, including contributions by countries, institutions, and authors, as well as journals, keywords, and co-cited references. Hydrogen therapy has shown significant efficacy in myocardial I/R injury, post-cardiac arrest syndrome, atherosclerosis and other cardiovascular diseases by regulating oxidative stress, inflammation and apoptosis. Investigation of the mechanisms of NLRP3 inflammasome and pyroptosis, the application of micro/nanomaterials in hydrogen therapy, and large-scale clinical research on hydrogen therapy for cardiovascular diseases are expected to be key research hotspots and represent the forefront of future investigations.

## Additional files:

***Additional file 1:*** *Retrieval strategies of hydrogen therapy for cardiovascular diseases.*

TS=("Cardiovascular Disease*" OR "Major Adverse Cardiac Event*" OR "Adverse Cardiac Event*" OR "Cardiac Event*" OR "Cardiovascular Abnormalit*" OR "Abnormalit*, Cardiovascular" OR "Heart Disease*" OR "Cardiac Disease*" OR "Cardiac Disorder*" OR "Heart Disorder*" OR "Heart Failure" OR "Cardiac Failure" OR "Heart Decompensation" OR "Heart Failure, Right-Sided" OR "Heart Failure, Right Sided" OR "Right-Sided Heart Failure" OR "Right Sided Heart Failure" OR "Myocardial Failure" OR "Congestive Heart Failure" OR "Heart Failure, Congestive" OR "Heart Failure, Left-Sided" OR "Heart Failure, Left Sided" OR "Left-Sided Heart Failure" OR "Left Sided Heart Failure" OR "Hypertension" OR "Blood Pressure*, High" OR "High Blood Pressure*" OR "Myocardial Ischemia" OR "Ischemic Preconditioning, Myocardial" OR "Myocardial Infarction" OR "Myocardial Reperfusion" OR "Myocardial Revascularization" OR "Myocardial Stunning" OR "Atherosclerosis" OR "Atherogenesis" OR "Myocardial ischemia reperfusion" OR "Heart Arrest" OR "Cardiopulmonary arrest" OR "Asystole" OR "Cardiac arrest" OR "Cardiomegaly" OR "Cardiac Hypertrophy" OR "Enlarged Heart" OR "Heart Enlargement" OR "Heart Hypertrophy") AND TS=("molecular hydrogen" OR "hydrogen-rich saline" OR "hydrogen rich saline" OR "hydrogen gas inhalation" OR "hydrogen-rich water" OR "hydrogen rich water" OR "hydrogen inhalation" OR "hydrogen gas" OR "hydrogen therapy" OR "hydrogen/oxygen therapy") AND LA=(English).

***Additional Table 1:*** *Top 10 co-cited references relevant to hydrogen therapy for cardiovascular diseases.*

## Data Availability

*All data relevant to the study are included in the article or uploaded as additional files.*

## References

[1] Li L, Li X, Zhang Z, Liu L, Zhou Y, Liu F (2020). Protective mechanism and clinical application of hydrogen in myocardial ischemia-reperfusion injury. Pak J Biol Sci.

[2] Ohsawa I, Ishikawa M, Takahashi K (2007). Hydrogen acts as a therapeutic antioxidant by selectively reducing cytotoxic oxygen radicals. Nat Med.

[3] Li H, Ma HY, Hua WL (2023). Trend of research on the medical use of molecular hydrogen: a bibliometric analysis. Med Gas Res.

[4] Tamura T, Hayashida K, Sano M (2016). Feasibility and safety of hydrogen gas inhalation for post-cardiac arrest syndrome - first-in-human pilot study. Circ J.

[5] Si Y, Tian H, Dong B (2021). Effects of hydrogen as adjuvant treatment for unstable angina. Exp Biol Med (Maywood).

[6] Ono H, Nishijima Y, Ohta S (2017). Hydrogen gas inhalation treatment in acute cerebral infarction: a randomized controlled clinical study on safety and neuroprotection. J Stroke Cerebrovasc Dis.

[7] Tao G, Zhang G, Chen W (2022). A randomized, placebo-controlled clinical trial of hydrogen/oxygen inhalation for non-alcoholic fatty liver disease. J Cell Mol Med.

[8] Nakao A, Toyoda Y, Sharma P, Evans M, Guthrie N (2010). Effectiveness of hydrogen rich water on antioxidant status of subjects with potential metabolic syndrome-an open label pilot study. J Clin Biochem Nutr.

[9] Zheng ZG, Sun WZ, Hu JY (2021). Hydrogen/oxygen therapy for the treatment of an acute exacerbation of chronic obstructive pulmonary disease: results of a multicenter, randomized, double-blind, parallel-group controlled trial. Respir Res.

[10] Sano M, Tamura T (2021). Hydrogen gas therapy: from preclinical studies to clinical trials. Curr Pharm Des.

[11] Shukla N, Merigó JM, Lammers T, Miranda L (2020). Half a century of computer methods and programs in biomedicine: A bibliometric analysis from 1970 to 2017. Comput Methods Programs Biomed.

[12] Goo B, Kim HN, Kim JH, Nam SS (2021). A bibliometric analysis of research on the treatment of facial nerve palsy. Medicine (Baltimore).

[13] Hausenloy DJ, Yellon DM (2013). Myocardial ischemia-reperfusion injury: a neglected therapeutic target. J Clin Invest.

[14] Hayashida K, Sano M, Ohsawa I (2008). Inhalation of hydrogen gas reduces infarct size in the rat model of myocardial ischemia-reperfusion injury. Biochem Biophys Res Commun.

[15] Li L, Liu T, Li X (2019). Protein chip and bioinformatic analyses of differentially expressed proteins involved in the effect of hydrogen-rich water on myocardial ischemia-reperfusion injury. Int J Med Sci.

[16] Li L, Li X, Zhang Z (2020). Effects of hydrogen-rich water on the PI3K/AKT signaling pathway in rats with myocardial ischemia-reperfusion injury. Curr Mol Med.

[17] Li L, Liu T, Liu L (2019). Effect of hydrogen-rich water on the Nrf2/ARE signaling pathway in rats with myocardial ischemia-reperfusion injury. J Bioenerg Biomembr.

[18] Zhang Y, Sun Q, He B, Xiao J, Wang Z, Sun X (2011). Anti-inflammatory effect of hydrogen-rich saline in a rat model of regional myocardial ischemia and reperfusion. Int J Cardiol.

[19] Pan S, Wang B, Yu M (2025). Hydrogen alleviates myocardial infarction by impeding apoptosis via ROS-mediated mitochondrial endogenous pathway. Free Radic Res.

[20] Nie C, Zou R, Pan S (2021). Hydrogen gas inhalation ameliorates cardiac remodelling and fibrosis by regulating NLRP3 inflammasome in myocardial infarction rats. J Cell Mol Med.

[21] Nie C, Ding X, A R (2021). Hydrogen gas inhalation alleviates myocardial ischemia-reperfusion injury by the inhibition of oxidative stress and NLRP3-mediated pyroptosis in rats. Life Sci.

[22] Wu F, Qiu Y, Ye G (2015). Treatment with hydrogen molecule attenuates cardiac dysfunction in streptozotocin-induced diabetic mice. Cardiovasc Pathol.

[23] Nie C, A R, Wang J (2023). Controlled release of hydrogen-carrying perfluorocarbons for ischemia myocardium-targeting (19) F MRI-guided reperfusion injury therapy. Adv Sci (Weinh).

[24] Katsumata Y, Sano F, Abe T (2017). The effects of hydrogen gas inhalation on adverse left ventricular remodeling after percutaneous coronary intervention for ST-elevated myocardial infarction - first pilot study in humans. Circ J.

[25] Atkins DL (2012). Cardiac arrest in children and young adults: we are making progress. Circulation.

[26] Stub D, Bernard S, Duffy SJ, Kaye DM (2011). Post cardiac arrest syndrome: a review of therapeutic strategies. Circulation.

[27] Hayashida K, Sano M, Kamimura N (2014). Hydrogen inhalation during normoxic resuscitation improves neurological outcome in a rat model of cardiac arrest independently of targeted temperature management. Circulation.

[28] Hayashida K, Sano M, Kamimura N (2012). H(2) gas improves functional outcome after cardiac arrest to an extent comparable to therapeutic hypothermia in a rat model. J Am Heart Assoc.

[29] Gong X, Fan X, Yin X (2022). Hydrogen therapy after resuscitation improves myocardial injury involving inhibition of autophagy in an asphyxial rat model of cardiac arrest. Exp Ther Med.

[30] Huo TT, Zeng Y, Liu XN (2014). Hydrogen-rich saline improves survival and neurological outcome after cardiac arrest and cardiopulmonary resuscitation in rats. Anesth Analg.

[31] Li Q, Yu P, Zeng Q (2016). Neuroprotective effect of hydrogen-rich saline in global cerebral ischemia/reperfusion rats: up-regulated tregs and down-regulated miR-21, miR-210 and NF-κB expression. Neurochem Res.

[32] Yin T, Becker LB, Choudhary RC (2021). Hydrogen gas with extracorporeal cardiopulmonary resuscitation improves survival after prolonged cardiac arrest in rats. J Transl Med.

[33] Tamura T, Suzuki M, Homma K, Sano M (2023). Efficacy of inhaled hydrogen on neurological outcome following brain ischaemia during post-cardiac arrest care (HYBRID II): a multi-centre, randomised, double-blind, placebo-controlled trial. EClinicalMedicine.

[34] Zheng H, Yu YS (2012). Chronic hydrogen-rich saline treatment attenuates vascular dysfunction in spontaneous hypertensive rats. Biochem Pharmacol.

[35] Liu L, Shi Q, Liu X, Li Y, Li X (2022). Attenuation of myocardial fibrosis using molecular hydrogen by inhibiting the TGF-β signaling pathway in spontaneous hypertensive rats. Am J Hypertens.

[36] Guan P, Lin XM, Yang SC (2019). Hydrogen gas reduces chronic intermittent hypoxia-induced hypertension by inhibiting sympathetic nerve activity and increasing vasodilator responses via the antioxidation. J Cell Biochem.

[37] Ji H, Sun H, Zhang Y (2024). Effectiveness and safety of hydrogen inhalation therapy as an additional treatment for hypertension in real-world practice: a retrospective, observational study in China. Front Cardiovasc Med.

[38] Kuropatkina T, Atiakshin D, Sychev F (2023). Hydrogen inhalation reduces lung inflammation and blood pressure in the experimental model of pulmonary hypertension in rats. Biomedicines.

[39] Kishimoto Y, Kato T, Ito M (2015). Hydrogen ameliorates pulmonary hypertension in rats by anti-inflammatory and antioxidant effects. J Thorac Cardiovasc Surg.

[40] Wang Y, Jing L, Zhao XM (2011). Protective effects of hydrogen-rich saline on monocrotaline-induced pulmonary hypertension in a rat model. Respir Res.

[41] Chi J, Li Z, Hong X (2018). Inhalation of hydrogen attenuates progression of chronic heart failure via suppression of oxidative stress and p53 related to apoptosis pathway in rats. Front Physiol.

[42] Grundy SM (2016). Metabolic syndrome update. Trends Cardiovasc Med.

[43] Ohsawa I, Nishimaki K, Yamagata K, Ishikawa M, Ohta S (2008). Consumption of hydrogen water prevents atherosclerosis in apolipoprotein E knockout mice. Biochem Biophys Res Commun.

[44] Liu B, Zhao M, Xue J, Gu Q, Zhang X, Qin S (2021). Hydrogen influences HDL-associated enzymes and reduces oxidized phospholipids levels in rats fed with a high-fat diet. Life Sci.

[45] Zong C, Song G, Yao S (2012). Administration of hydrogen-saturated saline decreases plasma low-density lipoprotein cholesterol levels and improves high-density lipoprotein function in high-fat diet-fed hamsters. Metabolism.

[46] Song G, Tian H, Liu J, Zhang H, Sun X, Qin S (2011). H2 inhibits TNF-α-induced lectin-like oxidized LDL receptor-1 expression by inhibiting nuclear factor κB activation in endothelial cells. Biotechnol Lett.

[47] Song G, Lin Q, Zhao H (2015). Hydrogen activates ATP-binding cassette transporter A1-dependent efflux ex vivo and improves high-density lipoprotein function in patients with hypercholesterolemia: a double-blinded, randomized, and placebo-controlled trial. J Clin Endocrinol Metab.

[48] Song G, Li M, Sang H (2013). Hydrogen-rich water decreases serum LDL-cholesterol levels and improves HDL function in patients with potential metabolic syndrome. J Lipid Res.

[49] LeBaron TW, Singh RB, Fatima G (2020). The effects of 24-week, high-concentration hydrogen-rich water on body composition, blood lipid profiles and inflammation biomarkers in men and women with metabolic syndrome: a randomized controlled trial. Diabetes Metab Syndr Obes.

[50] Zhou G, Goshi E, He Q (2019). Micro/nanomaterials-augmented hydrogen therapy. Adv Healthc Mater.

[51] He Y, Zhang B, Chen Y (2017). Image-guided hydrogen gas delivery for protection from myocardial ischemia-reperfusion injury via microbubbles. ACS Appl Mater Interfaces.

